# Thermotolerance Divergence Revealed by the Physiological and Molecular Responses in Two Oyster Subspecies of *Crassostrea gigas* in China

**DOI:** 10.3389/fphys.2019.01137

**Published:** 2019-09-10

**Authors:** Hamze Ghaffari, Wei Wang, Ao Li, Guofan Zhang, Li Li

**Affiliations:** ^1^Key Laboratory of Experimental Marine Biology, Institute of Oceanology, Chinese Academy of Sciences, Qingdao, China; ^2^University of Chinese Academy of Sciences, Beijing, China; ^3^National and Local Joint Engineering Key Laboratory of Ecological Mariculture, Institute of Oceanology, Chinese Academy of Sciences, Qingdao, China; ^4^Laboratory for Marine Biology and Biotechnology, Qingdao National Laboratory for Marine Science and Technology, Qingdao, China; ^5^Center for Ocean Mega-Science, Chinese Academy of Sciences, Qingdao, China; ^6^Laboratory for Marine Fisheries Science and Food Production Processes, Qingdao National Laboratory for Marine Science and Technology, Qingdao, China

**Keywords:** thermal tolerance, heart rate, metabolism, physiological performance, adaptive variation

## Abstract

Investigating the physiological mechanisms of closely related species that exhibit distinct geographic distributions and thermal niches is essential for understanding their thermal tolerance capacities and local adaptations in view of climate warming. The variations in upper thermal limits (LT_50_) under acute heat shock and cardiac activity, standard metabolic rate (SMR), anaerobic metabolite production and molecular responses (expression of molecular chaperones and glycolysis metabolism genes) under increasing temperatures in two oyster subspecies were studied. The populations of two oyster subspecies, *Crassostrea gigas gigas* and *C. gigas angulata*, exhibit different latitudinal distributions along the northern and southern coastlines of China, respectively, which experience different environmental conditions. The LT_50_ was significantly higher, by ∼1°C, in the southern than in the northern oysters. In both subspecies, temperature increases had powerful effects on heart rate, SMR and gene expression. The southern oysters had the highest Arrhenius breakpoint temperatures for heart rate (31.4 ± 0.17°C) and SMR (33.09°C), whereas the heart rate (28.86 ± 0.3°C) and SMR (29.22°C) of the northern oysters were lower. The same patterns were observed for the *Q*_10_ coefficients. More thermal sensitivity was observed in the northern oysters than in their southern counterparts, as the heat-shock proteins (HSPs) in the northern oysters were expressed first and had a higher induction at a lower temperature than those of southern oysters. Furthermore, different expression patterns of energetic metabolism genes (*HK, PK*, and *PEPCK*) were observed. In the northern oysters, increasing anaerobic glycolysis genes (*PEPCK*) and end products (succinate) were found at 36–43°C, indicating a transition from aerobic to anaerobic metabolism and a lower aerobic scope compared with the southern oysters. These two subspecies experience different environmental conditions, and their physiological performances suggested species-specific thermal tolerance windows in which the southern oysters, with mild physiological flexibility, had a higher potential capability to withstand heat stress. Overall, our results indicate that comparing and unifying physiological and molecular mechanisms can provide a framework for understanding the likely effects of global warming on marine ectotherms in intertidal regions.

## Introduction

Temperature is one of the major contemporary drivers of worldwide patterns of biodiversity that influences intertidal animal biogeographic distributions through biochemical, physiological and ecological settings, leading to possible evolutionary outcomes (Somero, 2012; Stuart-Smith et al., 2017). According to modeled climate predictions of a temperature increase of 1.5–5°C by 2100 (Pachauri et al., 2014), increasing temperatures may lead to challenges in survival and performance in intertidal organisms (Madeira et al., 2012; Sinclair et al., 2016). Understanding the role of temperature in establishing fine-scale patterns of thermal tolerance in closely related species can provide additional insights into the nature of adaptive variation in thermal tolerance (Sanford and Kelly, 2011). The relationship between distinctive zonation patterns and thermal tolerance has been indicated in intraspecific Pacific oyster (Li A. et al., 2018) as well as mussels (*Mytilus* species) (Halpin et al., 2004), snails (Dong et al., 2017), and limpet species (Prusina et al., 2014). Moreover, latitudinal patterns of variation in intra- and interspecific comparisons of ectotherms, such as fishes (Fangue et al., 2006), crabs (Gaitan-Espitia et al., 2017), mussels (Tagliarolo and McQuaid, 2015), and oysters (Li A. et al., 2017; Li L. et al., 2018) have been documented. These data often reflect the correlation of environmental factors with variation in the distribution patterns of organisms over temporal and spatial scales (Stuart-Smith et al., 2017).

Although studies on geographical variations in species have found thermal adaptation over a latitudinal gradient in intertidal regions (Yampolsky et al., 2014; Li L. et al., 2018), generally little evidence has been provided about the role of adaptation to thermal tolerance in oyster subspecies along the northern and southern coasts of China. Among sessile bivalves, oysters serve as experimental models for studies on physiological limitations in relation to population distribution patterns and biological responses to global change (Zhang et al., 2012; Bayne, 2017). Along China’s coast, *Crassostrea gigas gigas* (Thunberg, 1793) and *C. gigas angulata* (Lamarck, 1819) have colonized and have latitudinal distributions along the northern and southern intertidal habitats, respectively (Wang et al., 2010); as such, these oysters experience different environmental conditions. Phylogenic studies indicated divergence near 2.0–3.6 Mya, suggesting the presence of two subspecies (Ren et al., 2016).

A relationship between thermal limits and aerobic performance was proposed in the “oxygen- and capacity-limited thermal tolerance” (OCLTT) theory, in which a decreased capacity to perform aerobically at higher temperatures is considered to be the key physiological mechanism determining the response of many marine species to global warming (Pörtner et al., 2006; Pörtner, 2010). The limited capacities of the circulatory and ventilatory functions of aquatic ectotherms to compensate for the increased oxygen demand at higher temperatures cause a decline in aerobic scope and set the boundaries of whole-organism thermal tolerance (Kassahn et al., 2009; Sokolova et al., 2012). Accordingly, species that can maintain their aerobic capacity at warmer temperatures have a higher thermal tolerance and are thereby predicted to persist longer than species that encounter a reduction in aerobic function as temperature increases (Pörtner et al., 2012; Dowd et al., 2015).

Measuring the temperature that is lethal to 50% (LT_50_) of animals that are exposed to thermal stress provides a useful index of thermotolerance (Stillman, 2002; Angilletta, 2009). For instance, the negative relationship between the experimental rate of change in temperature and upper thermal tolerance was shown in Antarctic bivalves and oysters (Rajagopal et al., 2005; Pörtner et al., 2012). In addition, heart performance is assumed to be a reliable indicator of physiological rates in mollusks and contributes to establishing critical temperatures in many intertidal species (Bayne, 2017). In this context, comparative studies of heart rates in congeneric mussel species indicated that the critical temperature at which the heart rate collapsed was 4°C higher in warm-adapted mussels than in their cold-adapted counterparts (Somero, 2012). Moreover, the impact of temperature stress on oyster spat heart rates suggests that spat provides a model for conserved mechanisms of cardiac physiology (Domnik et al., 2016). Furthermore, the specific expression of heat-shock proteins (HSPs) in species inhabiting different latitudes may reflect their thermal adaptation to their environments (Hochachka and Somero, 2002; Tomanek, 2014). For example, variation in HSP expression patterns based on the different thermal niches of species has been indicated in congeneric oysters and snails, as the first and maximum induction HSPs were found at a higher temperature in species that inhabited thermally stresful environments (Tomanek, 2008; Li A. et al., 2019). To investigate the effects of environmental conditions on the thermotolerances of two oyster subspecies along the Chinese coastline, we defined the thermal performances for several physiological and molecular parameters. We examined the hypothesis that geographic variation in the phenotypic variables of *C. gigas gigas* and *C. gigas angulata* are driven by their thermotolerances and physiological capacities. To achieve this goal, the LT_50_, heart rate, standard metabolic rate, anaerobic end-product accumulation and expression of molecular chaperones (HSPs) with metabolic-related genes were quantified.

## Materials and Methods

### Collection Sites and Animal Maintenance

**FIGURE 1:**
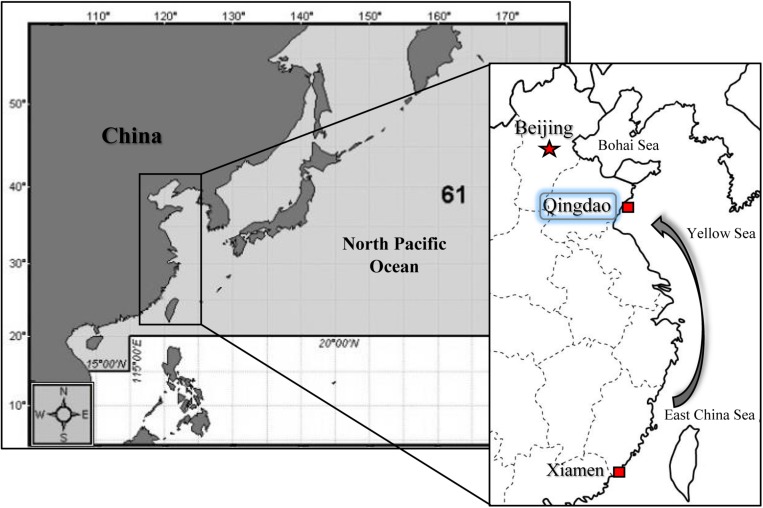
Sampling locations in North of China (Qingdao; QD) for *Crassostrea gigas gigas* and South of China (Xiamen; XM) for *C. gigas angulata.* Samples from the South were transported to Qingdao and both subspecies were acclimated in the same condition before experiment.

The cultured of two oyster subspecies, *C. gigas gigas* and *C. gigas angulata*, were collected from Qingdao, China (QD; 35°44′ N), and Xiamen, China (XM; 24°33′ N), respectively (Figure 1). The first collection was performed in the middle of autumn 2017 for the thermal tolerance experiment, and the second was performed in the early winter of 2018 for the physiological experiment. The oysters that were collected from Xiamen were sent to Qingdao and placed in aerated tanks. The oysters were scrubbed to remove extra attached particles and organisms and acclimated for 15 days in aerated tanks; they were fed every 2 days with commercial spirulina powder and maintained under a constant dark:light regime (12 h:12 h). The oysters were maintained in these conditions to attempt to remove the possible influence of any environmental cues (e.g., thermal variation, air exposure, and transportation). The shell lengths and widths of the selected oysters were measured with a Vernier calipers (mm). After drying the shells with blotting paper, the whole weight was measured (0.01 g). In addition, to assess environmental variation in temperature, the monthly average sea surface temperature (SST) over the last 19 years, from 2000 to 2018, was recorded using satellite remote sensing at Qingdao and Xiamen^1^.

### LT_50_ Determination and Thermal Tolerance

Adult oysters, *C. gigas gigas* (shell height 81.91 ± 0.43 mm, mean ± SE) and *C. gigas angulata* (80.85 ± 0.57 mm), were used to obtain basic information on the thermal tolerance ranges and LT_50_ values of theses subspecies after heat shock under laboratory conditions. In this part of the experiment, short-term (acute) heat treatment was used, and all experiments were repeated three times, each using 20 oysters per treatment. In the first step to determine the LT_50_, oysters were exposed to temperatures from 25 to 50°C in increments of 5°C. Two-step heat shock with two water baths was used to minimize the decrease in water temperature when the oysters were immersed. The oysters were immersed for 10 s in a water bath set at the desired temperature. The oysters were then immediately transferred to the second water bath (Tianheng, model 0530, China) set at the desired temperature. The oysters were maintained at this temperature for 1 h and subsequently returned to the ambient temperature for 7 days. Mortality was then assessed based on whether the oysters could keep their valves closed tightly when stimulated mechanically. During the first step of exposure, which was described above, the temperature range of highest mortality was detected. To assess the temperature that induced LT_50_, the oysters were exposed to a narrower temperature range, an increase of 0.5°C, for 1 h, as described above. Basic laboratory measurements of temperature-induced mortality contributed to the determination of the sublethal, lethal and LT_50__,_ limits that were the basis for subsequent studies on thermal tolerance in these two oyster subspecies.

### Physiological Studies

In this research, the SMR, heart rate, Arrhenius model and anaerobic end products were measured to provide a detailed description of physiological fitness in relation to heat stress.

#### SMR and Heart Rate Assays

The SMRs of the oysters were measured with a closed respiration system. A closed respiration glass chamber was placed in a temperature-controlled tank connected to a circulating precision water bath (Tianheng, model 0530, China). The control temperature was maintained at 17°C, and the experimental temperatures were adjusted to six acute points (20, 24, 28, 32, 36, and 40), with an increase rate of 5°C per hour. This rate was the same for the subsequent treatments. These temperatures were selected according to the annual temperature ranges, the increase predicted for the year 2100 by an Intergovernmental Panel on Climate Change (IPCC) scenario and the temperature changes that oysters experience during the tidal cycle in their habitats. After acclimation, the *C. gigas gigas* (shell height 64.73 ± 0.29 mm, mean ± SE) and *C. gigas angulata* (59.06 ± 0.31 mm) specimens were carefully cleaned with 95% ethanol to remove attached organisms and extra particles. Magnetic stirrers with an embedded small magnet in the respiration chamber were used to circulate seawater constantly. A non-invasive oxygen microsensor (needle type) and a temperature probe (PreSens GmbH, Germany^2^) were fitted into the lid, and the probes were then connected to an oxygen meter (Microx; PreSens) to measure the oxygen concentration and temperature every 1 s. According to the manufacturer’s instructions, the oxygen sensors were calibrated using a two-point calibration (0 and 100% air saturation) to create similar temperature and salinity conditions before each trial. The oysters were fasted for 24 h before the O_2_ concentration was measured. Individual oysters were paced directly into the 750 mL chamber that was adjusted to the ambient temperature and warmed to reach the desired temperature. The chamber was then closed, and the measurements began. At the end of each experiment, the oysters were removed from the respiration chamber and the shell-free dry weight was determined by drying the oyster flesh at 80°C for 24 h. The SMR was calculated as follows:

(1) $$S\,M\,R=\left(\frac{V_{r}\times \Delta \,C_{w}\,O_{2}}{\Delta \,t\times M}\right)$$

where *SMR* is the normalized oxygen consumption per 1 g of dry mass (mg O_2_ h^–1^ g^–1^ dry mass), *V*r is the volume of the respirometry chamber minus the volume of an oyster (L), Δ*C*_w_O_2_ is the difference in the oxygen concentration during the measurement (mg O_2_ L^–1^), Δ*t* is the measuring time (h) and *M* is the dry mass (g).

The heart rates of oysters of similar size [shell height: 86.77 ± 2.24 mm (*C. gigas gigas*); 82.09 ± 3.65 mm (*C. gigas angulata*)] were measured via continuous simultaneous determination using a non-invasive technique (Depledge et al., 1996; Hellicar et al., 2015). A round hole less than 1 cm in diameter was drilled on the right shell valve on top of the pericardium while avoiding damage to the mantle tissue during the drilling process. An infrared sensor (CNY70) was fixed directly above the heart using Blue-Tack (Bostik, United Kingdom) and sealed with super glue (Krazy, Japan) to ensure that the integrity of the shell was maintained. The oysters were allowed to recover from drilling and sensor implantation for 12–24 h, and feeding was stopped 24 h before starting the measurements. The oysters were left undisturbed for 30 min to recover from handling and for the heart rate signal to stabilize before the recordings began. The reflected optical signal of the heart was measured via an IR diode detector and amplified and filtered using a data acquisition system (Powerlab 8/35, AD Instruments, Germany). The data were digitally recorded and analyzed using LabChart software (version 8.0, AD Instruments). After acclimation, the oysters were exposed to continuously increasing temperatures from 12 to 45°C using a circulating precision water bath (Tianheng, model 0530, China); the individuals were kept in aerated seawater, and the heart rates were recorded continuously. To ensure that the temperatures were stable, the water bath temperature was recorded every 30 s using a thermo-probe (PreSens, Germany). The heart rates (beats per min) of eight individuals of each subspecies were recorded every 10 min. To measure the heart rate, at least three heartbeat periods were counted within 10 min. Individual heart rates from the three counts were averaged and calculated per min.

#### Arrhenius Breakpoint Temperature (ABT), Arrhenius Activation Energy, and *Q*_10_ Determination

The ABT, the temperature at which respiration and the heart rate dramatically decrease (Parker et al., 2017), was indicated by an Arrhenius plot and calculated using regression analyses of the natural logarithm of the heart rates and SMRs against the absolute temperature (1000/T). This process generated two linear regressions that best fit the data, and the intersection point of these two lines was considered the ABT of each individual. The slope of the natural log (ln) from the linear regressions of the Arrhenius plot for the heart rates and SMRs, which increased with increasing temperature, were used to calculate the activation energy (*E*_a_, mean energy required for the enzyme-catalyzed biochemical reactions of metabolism), which was expressed in KJ mol^–1^. The *R*^2^ values for each treatment were represented as the mean ± SE. To evaluate potential differences in the thermal sensitivity of increasing the temperature by 10°C, the following *Q*_10_ equation (temperature coefficient) was used:

$$H\,e\,a\,r\,t\,r\,a\,t\,e\,Q_{10}={\left(H\,R_{2}/H\,R_{1}\right)}^{\left(10/T_{2}-T_{1}\right)}$$

$$S\,M\,R\,Q_{10}={\left(S\,M\,R_{2}/S\,M\,R_{1}\right)}^{\left(10/T_{2}-T_{1}\right)}$$

where *HR*_1_ and *SMR*_1_ are the measured heart rate and SMR at temperature 1 (*T*_1_,°C), respectively, and *HR*_2_ and *SMR*_2_ are the measured heart rate and SMR at temperature 2 (*T*_2_,°C), respectively (where *T*_1_ < *T*_2_).

#### Anaerobic End-Product Assay

The same rate of increasing temperature was used to determine the succinic and malic acid concentrations. Succinate is the most well-known anaerobic end-product and is an indicator of anaerobic metabolism (Michaelidis et al., 2005; Bayne, 2017). After preliminary acclimation, the *C. gigas gigas* (shell height: 87.15 ± 0.84 mm, mean ± SE) and *C. gigas angulata* (85.53 ± 0.64 mm) specimens were exposed to continuous increasing temperatures starting at 12°C to the target temperatures (22, 29, 36, 40, and 43°C) and kept at these temperatures for 1 h. The adductor muscles were immediately dissected and shock-frozen in liquid nitrogen and then preserved at −80°C before being lyophilized for 24 h. The dry tissue was ground into a powder with a grinder (Scientz-48) and weighed, and the sample powder (∼20 mg) was added to an excess volume (4×) of cold Trichloric acetic acid (TCA). The homogenate was centrifuged for 3 min at 14,000 × *g*, and the supernatant was neutralized by mixing with 4 volumes of 1:4 *n*-octylamine: 1,1,2-trichlortrifluorethane and centrifuged (2 min, 14,000 × *g*). The upper phase was collected, diluted 1:3 with deionized water, filtered through a 0.2 μm filter and measured with a capillary electrophoresis system (7100A, Agilent, United States). Succinate was separated with an eCAP capillary that was 60 cm in length and 50 mm in diameter (Nuoxin, Cangzhou, China) at a constant voltage of 30 kV and a temperature of 25°C and detected at 214 nm. The separating buffer (pH 8.6) consisted of 30 mM Tris, 4 mM trimesic acid and 0.015% (W/V) HDB.

### HSPs and Metabolic-Related Gene Expression

As mentioned above, the oysters were exposed to continuously increasing temperatures (5°C per h) from 12°C to the target temperatures (22, 29, 36, 40, and 43°C) and kept at these temperatures for 1 h to determine the temperature sensitivity and expression of metabolic and HSP family genes. At 12°C (control) and the target temperatures, the oyster gills were immediately dissected and frozen in liquid nitrogen and then stored at −80°C prior to the estimation of gene expression.

Total RNA was isolated from ∼20 mg of frozen gill tissue with a RNA Pure Tissue Kit (Tiangen, Beijing, China) according to the manufacturer’s instructions. The RNA quality and concentrations were assessed via gel electrophoresis and a Nanodrop 2000 spectrophotometer, respectively. After this step, equal amounts (1 μg) of RNA from four oysters were pooled together at each target temperature of each subspecies to avoid the potential for exceptionally high expression of a given gene in one sample. First strand cDNA was obtained using a PrimeScript RT reagent Kit (Takara, Japan) on 1 μg of total RNA following the manufacturer’s instructions. The expression of mRNA for the target genes was determined using an Applied Biosystems 7500 Real-Time PCR System (Applied Biosystems, United States). In this study, nine HSPs and three metabolic-related genes were used for assessing mRNA expression using real-time PCR. Elongation factor 1 alpha (*EF1*α) was used as an internal control based on its low expressional variability during acute heat stress (Li A. et al., 2017). The slopes of standard curves for each primer pair from serial dilutions of cDNA were used to determine the amplification efficiency in both subspecies (Ståhlberg et al., 2003; Li A. et al., 2017) (Supplementary Table 1).

Quantitative real-time PCR amplifications were carried out in triplicate in a final volume of 20 μl that contained 10 μl of SYBR Green 2× Supermix (Takara), 5.8 μl of DEPC H_2_O, 0.4 μL of each forward and reverse primer and ROX Dye II and 3 μl of 20× diluted cDNA template. All q-RT-PCR assay runs began with a 30 s activation of DNA polymerase at 95°C followed by 40 cycles of 5 s at 95°C and 30 s at 60°C. Dissociation curve analysis of the amplicons was conducted as follows: 15 s at 95°C, 1 min at 60°C, 30 s at 95°C, and 15 s at 60°C. Differences between the CT values of the amplified genes and EF (ΔCT) were calculated, and the comparative method of relative basal (12°C) and induced transcripts (22, 29, 36, 40, and 43°C) were determined with the CT method (2^–ΔΔCT^) to analyze the expression level of the target genes.

### Data Analysis

The normality of the data distributions was checked using a Shapiro–Wilk’s test, and the assumption of homoscedasticity of variances was tested using Levene’s test. Logistic and polynomial regressions were used to model the LT_50_ and any significant differences. To analyze the SMR, anaerobic end products and gene expression at different temperatures, subspecies and their interaction, two-way ANOVA (fixed factors: subspecies and temperatures) was carried out followed by *post hoc* tests to determine the differences for each subspecies. Differences in the slopes of the Arrhenius plots, ABTs and *Q*_10_ values among these two oyster subspecies were tested by Student’s *t*-test. Analysis of covariance (ANCOVA) was used to test for any inter-individual differences in heart rate and anaerobic end products for each subspecies. Non-parametric Kruskal–Wallis tests were performed because the data did not have a normal distribution. All statistical analyses were carried out with IBM SPSS Statistics 25.0.

## Results

### Air and Seawater Temperature

The annual average air temperature from 2015 to 2016 and SST were >8 and >7°C, respectively, and there were clear differences between the two native habitats (Li A. et al., 2017). The monthly average SST from satellite remote sensing revealed distinct geographic patterns in our study area from 2000 to 2018. In general, *C. gigas gigas* experienced the most seasonal variations in seawater temperature, from 5°C in winter to 26°C in summer. These temperatures were 15°C in winter and 28°C in summer for *C. gigas angulata*, with annual averages of 15.3 and 21.3°C in the northern (Qingdao; QD) and southern (Xiamen; XM) sampling areas, respectively. These data indicated significant differences in the average seawater temperature in each month between the two sampling sites (Supplementary Figure 1). During the summer and according to the weather data from both sites, the diurnal tide cycle and sun radiation indicated that intertidal species face extreme temperatures. Therefore, these different climatic conditions on the northern and southern coasts of China provide unique environments for both subspecies.

### LT_50_ Determination in the Northern and Southern Oyster Subspecies

**FIGURE 2:**
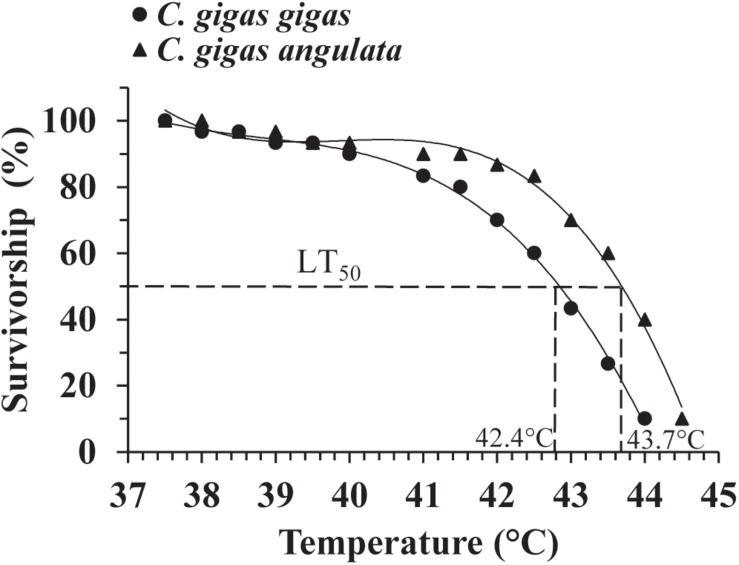
Upper LT_50_ in *Crassostrea gigas gigas* (circles) and *C. gigas angulata* (triangles). Data were fit with third order regression for modeling LT_50_.

A preliminary test of the thermal tolerance limits indicated that 100% mortality occurred at temperatures greater than 40°C in both subspecies (Supplementary Figure 2); accordingly, a narrower thermal range of 35–45°C was used to determine the LT_50_. During this trial, no mortality was observed when both subspecies were exposed for 1 h to temperatures below 37°C. Direct heat shock greater than 44°C, however, always led to 100% mortality. The LT_50_ result described above indicated that 37 and 44°C were the sublethal (100% survived) and lethal (100% died) temperatures, respectively. The survival data were fit to a third-order regression for each oyster subspecies, and logistic regression (LGR) predicted the LT_50_ to be 42.4°C for *C. gigas gigas* and 43.7°C for *C. gigas angulata* (Figure 2). The LT_50_ regression models for both oyster subspecies were highly significant (LGR, *P* < 0.001, *r*^2^ = 0.896 for *C. gigas gigas* and *P* < 0.001, *r*^2^ = 0.885 for *C. gigas angulata*) (Supplementary Table 2).

### SMR and Heart Rate Assay

**FIGURE 3:**
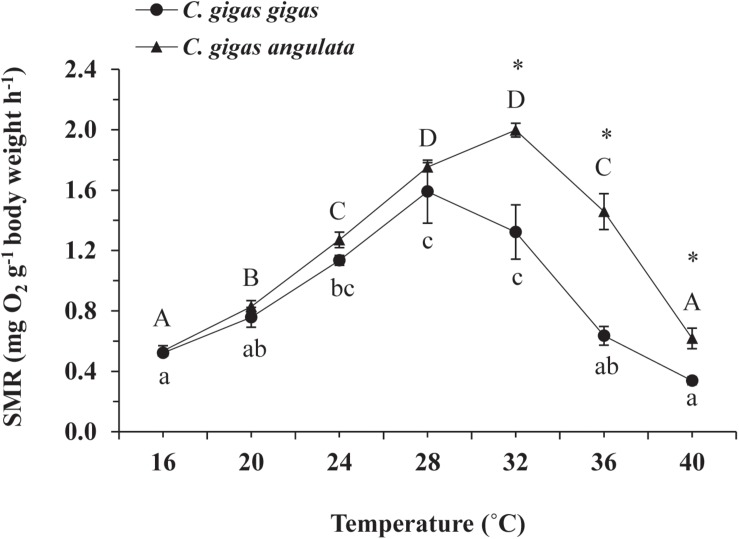
Effect of increasing temperature from 17°C on the standard metabolic rate (SMR) of *Crassostrea gigas gigas* and *C. gigas angulata* (mean ± SE; *n* = 4 for each subspecies). Lowercase and capital letters indicates significant differences between temperatures but within a subspecies and an asterisk indicates a significant differences between subspecies within a temperature (*P* < 0.05 for all significant comparisons).

The effect of acute temperature increases demonstrated important differences in the SMRs between these two subspecies, where the SMR was greatest at 28°C for *C. gigas gigas* and 32°C for *C. gigas angulata* (Figure 3). Two-way ANOVA revealed that the SMR differed at different acute temperatures and in the different subspecies, with the interactive effect between these two factors (temperature and subspecies) being statistically significant (Table 1). In addition, *post hoc* Tukey analyses indicated that, with warming, the SMRs in both subspecies increased significantly at high temperatures, specifically in the range of 28–32°C. Moreover, a statistical comparison between the two subspecies revealed that the SMR was significantly higher in *C. gigas angulata* than in *C. gigas gigas* at 32, 36, and 40°C (*t*-test, *df* = 6, *P* < 0.01), with the southern species having a high metabolic rate. In *C. gigas angulata*, the SMR increased with temperature up to 32°C and then gradually decreased at 36 and 40°C. A similar pattern was found in *C. gigas gigas*; the SMR increased to a maximum level at 28°C and decreased at 32, 36, and 40°C (Figure 3).

**TABLE 1:** Two-way ANOVA analysis of standard metabolic rate (SMR) and anaerobic end-products of *Crassostrea gigas gigas* and *C. gigas angulata* under different temperature treatments (^∗∗∗^*P* < 0.0005, ^∗∗^*P* < 0.005, ^∗^*P* < 0.05).

		**Factors**	**SS**	**df**	**MS**	***F***	***P*-value**	**Significant**
Standard metabolic rate		Subspecies	1.456	1	1.456	44.850	3.949E-08	^∗∗∗^
		Temperature	11.802	6	1.967	60.587	4.292E-19	^∗∗∗^
		Subspecies × temperature	1.112	6	0.185	5.710	0.0002	^∗∗∗^
Anaerobic end-products	Succinate	Subspecies	36.339	1	36.339	21.439	2.805E-05	^∗∗∗^
		Temperature	723.851	5	144.770	85.410	1.050E-22	^∗∗∗^
		Subspecies × temperature	78.008	5	15.602	9.204	3.415E-06	^∗∗∗^
	Malate	Subspecies	396.590	1	396.590	58.912	6.836E-10	^∗∗∗^
		Temperature	1814.129	5	362.826	53.897	1.535E-18	^∗∗∗^
		Subspecies × temperature	516.098	5	103.220	15.333	5.384E-09	^∗∗∗^

**FIGURE 4:**
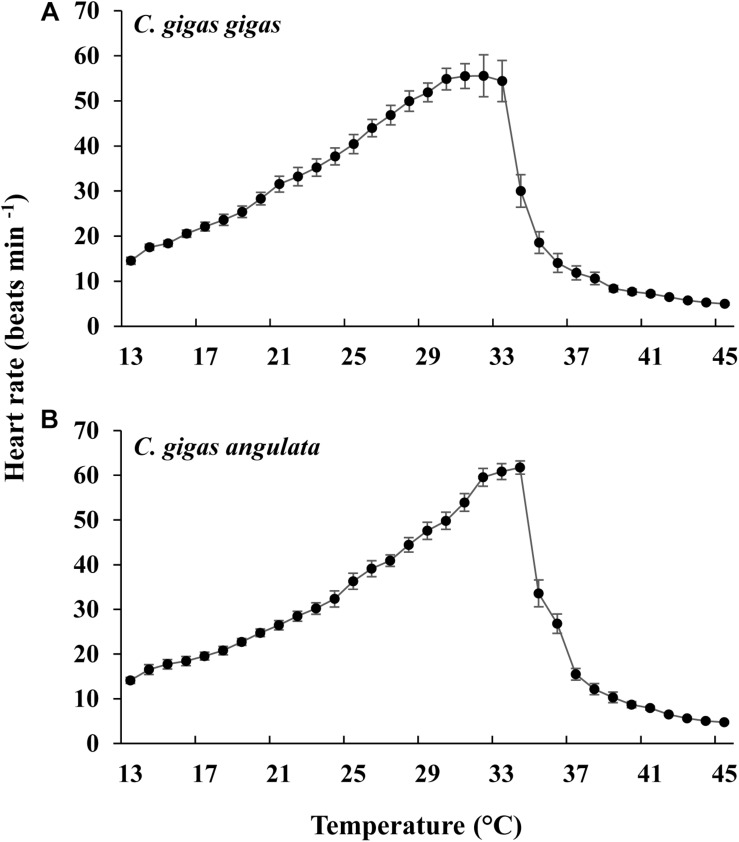
Effect of increasing temperature from 12°C on the heart rate of *Crassostrea gigas gigas* **(A)** and *C. gigas angulata* **(B)** (mean ± SE; *n* = 8 for each subspecies).

As the temperature increased, the heart rate increased, being highest at 31°C in *C. gigas gigas* and 34°C in *C. gigas angulata*. There were no differences in the heart rate between these two subspecies at 13°C, which was the ambient temperature at the beginning of the recording (*t*-test, *df* = 14, *P* = 0.98); however, the heart rate of *C. gigas angulata* at 34°C was significantly higher than that of *C. gigas gigas* (*t*-test, *df* = 14, *P* < 0.001). After this period with continuously increasing temperature, both subspecies experienced a steady rhythm, and the heart rate decreased dramatically (Figure 4). Throughout the experiment, *C. gigas gigas* had a faster heart rate (1.8 ± 0.4 Hz, mean ± SD) than *C. gigas angulata* (1.6 ± 0.3 Hz, mean ± SD), which is a physiological behavior (Supplementary Figure 3).

### ABT, *Q*_10_, and Anaerobic End-Product

**FIGURE 5:**
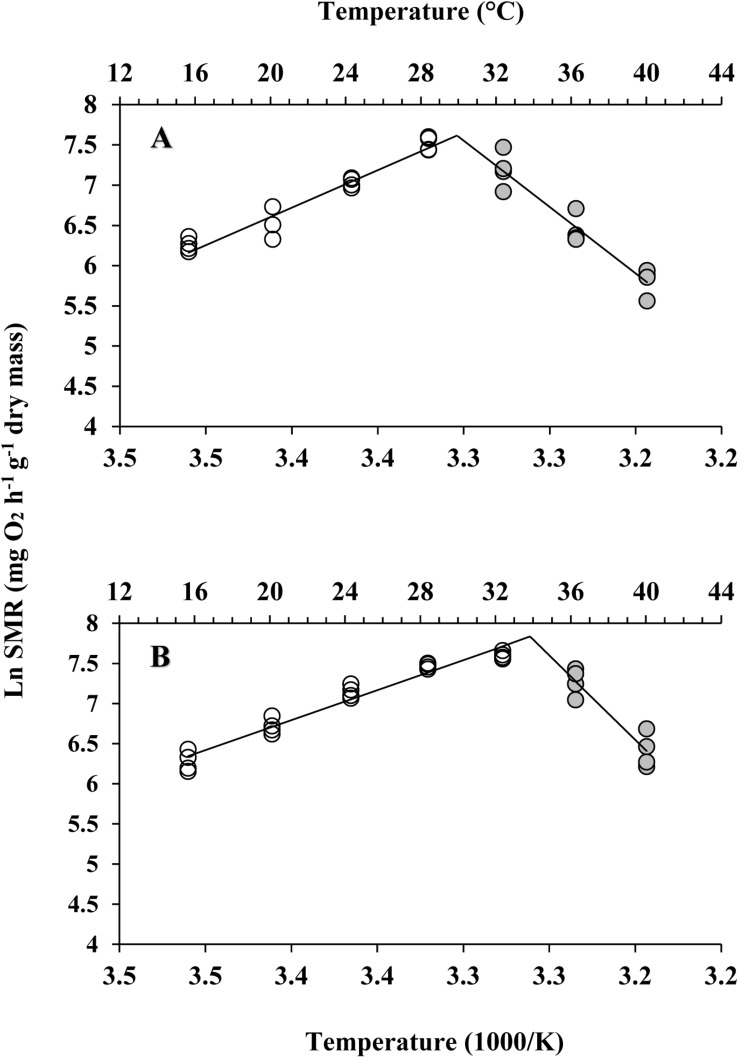
Arrhenius break temperatures (ABT) of standard metabolic rate (SMR) for *Crassostrea gigas gigas* **(A)** and *C. gigas angulata* **(B)** (*N* = 4 for each subspecies).

**FIGURE 6:**
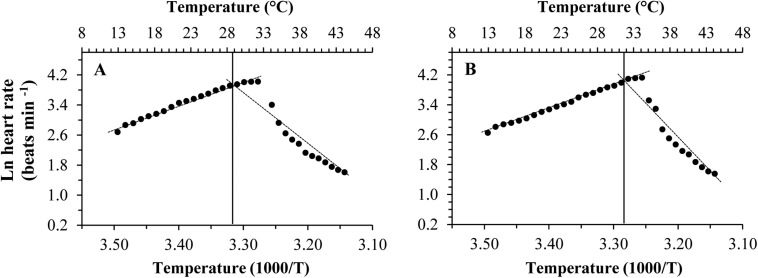
Arrhenius break temperatures (ABT) of heart rates for *Crassostrea gigas gigas* **(A)** and *C. gigas angulata* **(B)** (*N* = 8).

Different patterns of SMR in relation to warming were observed in these two subspecies (Figure 5). Linear regression of the Arrhenius plot for the temperature range over which the SMR increased with increasing temperature showed significant differences in the slope, with *C. gigas gigas* having a higher slope than the other subspecies (*t*-test, *df* = 6, *P* < 0.05). The ABTs were 33.09 and 29.22°C for *C. gigas angulata* and *C. gigas gigas*, respectively, and this temperature was significantly different between these two subspecies (*t*-test, *df* = 6, *P* < 0.05). The *Q*_10_ ratio was similar (∼2.5) in the increasing temperature range from 16 to 28°C, while above these temperatures, the *Q*_10_ ratio dropped to 1.38 and 0.65 in *C. gigas angulata* and *C. gigas gigas*, respectively. The *Q*_10_ value of the *C. gigas gigas* subspecies indicated that the species was completely temperature-independent at temperatures from 28 to 32°C (Table 2). Similar to the SMR, the Arrhenius plots of heart rate (Figure 6) and its two-stage regression indicated that the ABTs for these two subspecies were significantly different (ANCOVA, *P* = 0.0001). The ABT of *C. gigas angulata* was higher (31.42 ± 0.17°C, mean ± SE) than that of *C. gigas gigas* (28.86 ± 0.3°C) (*t*-test, *df* = 14, *P* < 0.001), but the slopes of the Arrhenius plots were not significantly different between the two subspecies (*t*-test, *df* = 14, *P* > 0.05) (Table 3). The calculated *Q*_10_ ratios indicated that the heart rate increased with increasing temperature in the range from 13 to 23°C in both subspecies, and the values were similar. In the second temperature range (23–33°C), the *Q*_10_ values were significantly different between these two subspecies (*t*-test, *df* = 14, *P* > 0.001). In *C. gigas angulata*, the *Q*_10_ value remained unchanged and high (2.03 ± 0.06, mean ± SE), whereas in *C. gigas gigas*, the value fell from 2.4 to 1.5, revealing a decline in metabolic rate. Over these temperatures, the *Q*_10_ values became < 1, indicating that temperature change likely negatively impacted the metabolic rate (Table 3).

**TABLE 2:** Arrhenius plot regression parameters of *Crassostrea gigas gigas* and *C. gigas angulata* (*E*_a_: activation energy, J mol^–1^; *R*: ideal gas constant, 8.31 J K^–1^ mol^–1^), Arrhenius break point temperature (ABT) and *Q*_10_ values for standard metabolic rate exposed to increasing temperature (20–40°C) (*Q*_10_ values <1 are shown in italic).

	**ln (a)**	***E*_a/_*R***	**ABT**	***Q*_10_**					
				
				**16–20°C**	**20–24°C**	**24–28°C**	**28–32°C**	**32–36°C**	**36–40°C**
*C. gigas gigas*	38.6 ± 2.25	9.37 ± 0.66	29.22	2.53	2.74	2.32	*0.63*	*0.15*	*0.2*
*C. gigas angulata*	32.52 ± 0.58	7.56 ± 0.17	33.09	2.97	2.91	2.23	1.38	*0.45*	*0.11*

**TABLE 3:** Arrhenius plot regression parameters (mean ± SE) of *Crassostrea gigas gigas* and *C. gigas angulata* (a: normalization constant; *E*_a_: activation energy, J mol^–1^; *R*: ideal gas constant, 8.31 J K^–1^ mol^–1^), *R*^2^ for the linear regression of ln heart rate on the inverse of absolute temperature and Arrhenius break point temperature (ABT) and *Q*_10_ values for heart rate exposed to increasing temperature (13–43°C) (*Q*_10_ values <1 are shown in italic).

	**ln (a)**	***E*_a/_*R***	***R*^2^**	**ABT**	***Q*_10_**		
					
					**13–23°C**	**23–33°C**	**33–43°C**
*C. gigas gigas*	24.99 ± 0.67	6.35 ± 0.19	0.97 ± 0.02	28.86 ± 0.30	2.42 ± 0.07	1.50 ± 0.10	*0.11* ± *0.07*
*C. gigas angulata*	24.64 ± 0.43	6.28 ± 0.13	0.98 ± 0.03	31.42 ± 0.17	2.16 ± 0.11	2.03 ± 0.06	*0.09* ± *0.05*

**FIGURE 7:**
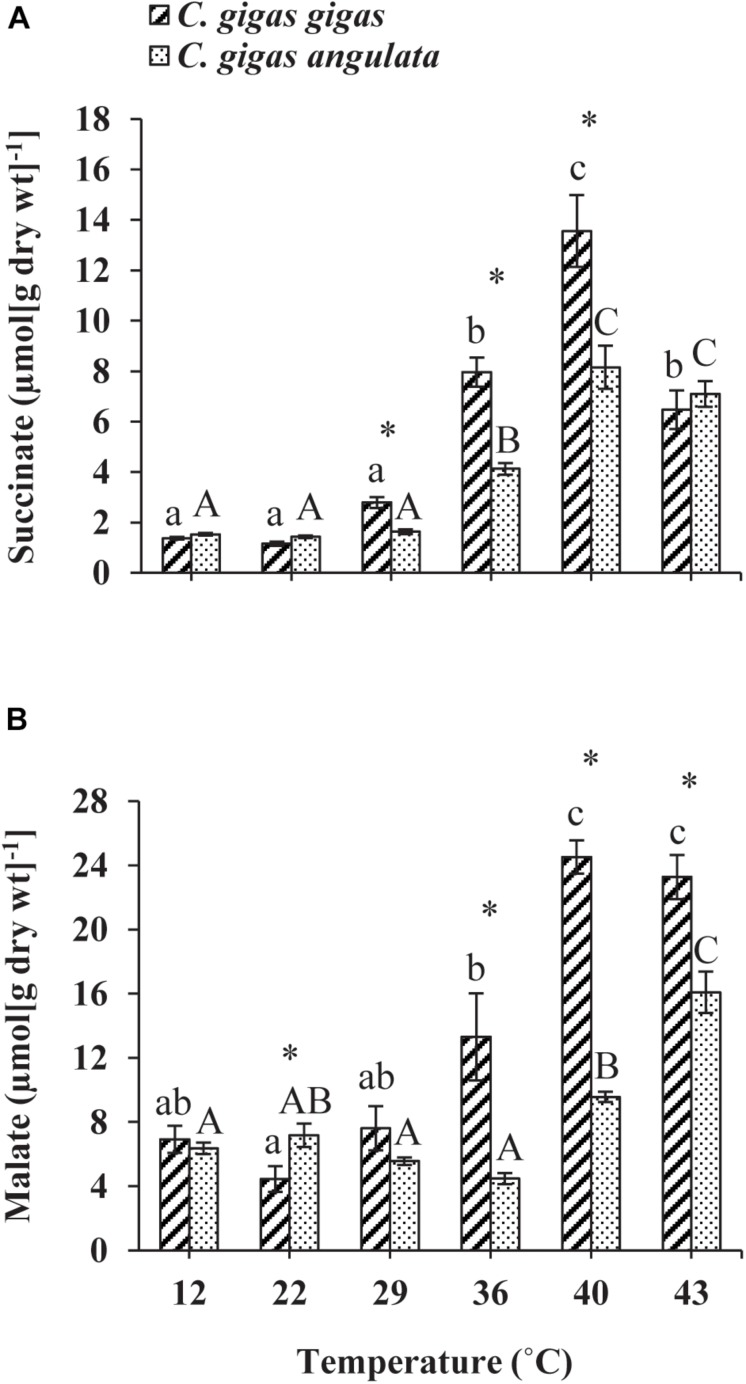
Anaerobic end-products **(A)** Succinate and **(B)** malate concentration in adductor muscle of *Crassostrea gigas gigas* and *C. gigas angulata* for 1 h after exposure to different temperature regimes (22, 29, 36, 40, and 43°C). Lowercase and capital letters indicates significant differences between temperatures but within a subspecies and an asterisk indicates a significant differences between subspecies within a temperature (*P* < 0.05 for all significant comparisons).

The anaerobic capacities of succinate and malate production was recorded in the adductor muscle tissues of these two subspecies after being exposed to increasing temperatures from 12 to 43°C. Two-way ANOVA of the anaerobic metabolites showed that the concentrations of succinate and malate at different temperatures and subspecies and the interaction between these two factors (temperature and subspecies) differed significantly (Table 1). Although similar response patterns to high temperature and similar concentrations at 12°C were found for both subspecies, *C. gigas gigas* had a higher accumulation rate of succinate at 29 and 36°C and the highest value at 40°C (9.8-fold increase); in comparison, the highest value for *C. gigas angulata* was at 40°C (5.3-fold increase) (*t*-test, *df* = 8, *P* < 0.01). Similarly, the malate content increase was temperature-dependent and reached its highest contents at 40°C (3.5-fold increase) and 43°C (2.5-fold increase) in *C. gigas gigas* and *C. gigas angulata*, respectively (*t*-test, *df* = 8, *P* < 0.008). *Post hoc* tests on the different temperatures in both subspecies indicated differences (Tukey’s HSD, *P* < 0.05; Figure 7).

### HSPs and Metabolic-Related Gene Expression

**FIGURE 8:**
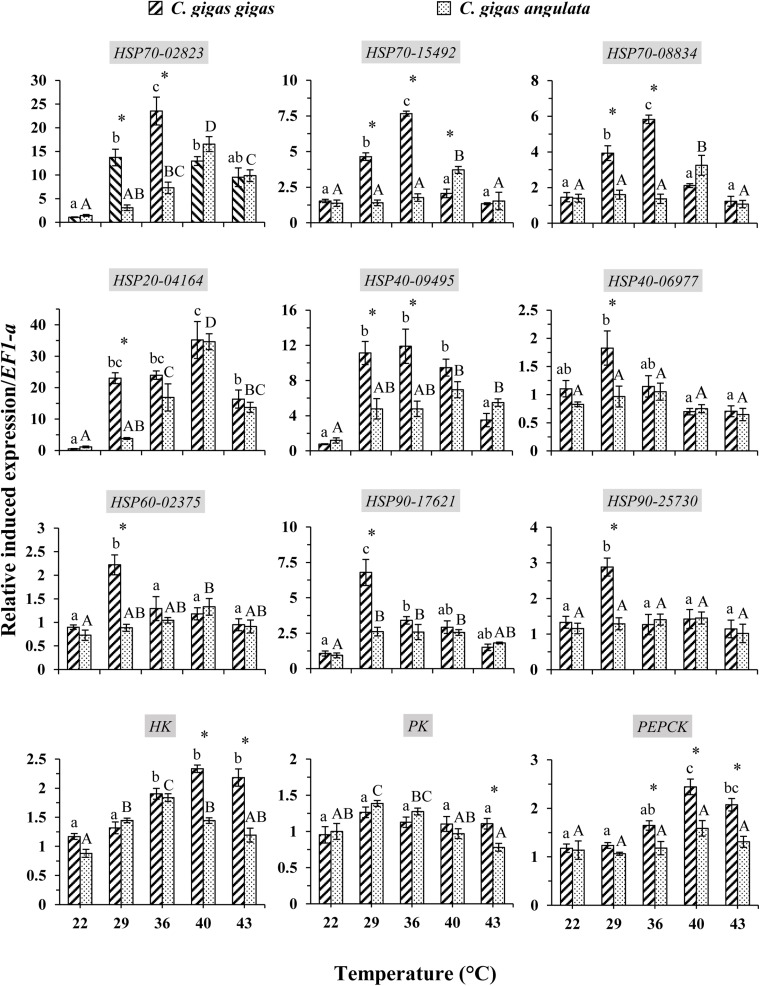
Induced expression of HSPs and metabolic-related genes in *Crassostrea gigas gigas* and *C. gigas angulata* for 1 h after exposure to different temperature regimes (22, 29, 36, 40, and 43°C). Lowercase and capital letters indicates significant differences between temperatures but within a subspecies and an asterisk indicates a significant differences between subspecies within a temperature (*P* < 0.05 for all significant comparisons).

These two subspecies showed differences in their mRNA expression patterns of inducible HSPs and three major metabolic genes (*HK*, *PK*, and *PEPCK*) (Figure 8). In general, in both subspecies, gene expression exhibited a similarly low level of mRNA in response to increasing temperature at 22°C, indicating minimum thermal stress at this temperature. When these subspecies were exposed to the high temperature, significant disparities were revealed in the mRNA levels between these two subspecies (Table 4). When comparing the HSP mRNA levels between these two subspecies, a high degree of upregulation at the beginning of heat stress was found in *C. gigas gigas*, indicating the higher sensitivity of this subspecies to heat shock, whereas *C. gigas angulata* exhibited a delay and more upregulation levels at a higher temperature (*t*-test, *df* = 4, *P* < 0.05). The HSP70s and *HSP40-09495* levels in both subspecies varied with temperature, with the highest upregulation at 36 and 40°C in *C. gigas gigas* and *C. gigas angulata*, respectively; then, their peak expression dropped to a low level at higher temperatures. The expression of *HSP20-04164* in *C. gigas gigas* showed a high degree of upregulation at 29°C compared with that of *C. gigas angulata*, although it became highest and similar at 40°C in both subspecies. The other selected HSPs, indicating *HSP40-06977*, *HSP60-02375*, *HSP90-17621* and *HSP90-25730*, expression reached a peak at 29°C in *C. gigas gigas*, while *C. gigas angulata* exhibited weak expression levels at all temperatures (Tukey’s HSD, *P* < 0.05). Moreover, two-way ANOVA of the metabolic responsive genes (*HK*, *PK*, and *PEPCK*) demonstrated a significant effect of temperature, subspecies and the interaction of the factors (temperature and subspecies), although *PK* expression did not present significant differences between these two subspecies (Table 5). *Post hoc* Tukey tests of gene expression at different temperatures in both subspecies revealed that in *C. gigas gigas*, *HK* and *PEPCK* expression increased at 36°C with the highest expression level at 40°C. In *C. gigas angulata*, *HK* also increased at 36°C but *PEPCK* showed a slight and non-significant increase. In addition, the mRNA levels of *HK* and *PEPCK* increased in *C. gigas gigas* as the temperature increased, while *PK* did not change when the temperature increased. In contrast, in *C. gigas angulata*, *HK* and *PK* were downregulated, and the expression of *PEPCK* did not change (Figure 8).

**TABLE 4:** Two-way ANOVA analysis of HSP genes expressions of *Crassostrea gigas gigas* and *C. gigas angulata* under different temperature treatments (^∗∗∗^*P* < 0.0005, ^∗∗^*P* < 0.005, ^∗^*P* < 0.05; ns, not significant).

	**Factors**	**SS**	**df**	**MS**	***F***	***P*-value**	**Significant**
	Subspecies	153.150	1	153.150	22.798	0.0001	^∗∗∗^
*HSP70-02823*	Temperature	785.033	4	196.258	29.215	4.237E-08	^∗∗∗^
	Subspecies × temperature	431.921	4	107.980	16.074	4.877E-06	^∗∗∗^
	Subspecies	16.676	1	16.676	69.210	6.335E-08	^∗∗∗^
*HSP70-15492*	Temperature	44.211	4	11.053	45.873	8.426E-10	^∗∗∗^
	Subspecies × temperature	55.258	4	13.814	57.334	1.125E-10	^∗∗∗^
	Subspecies	10.314	1	10.314	37.384	5.642E-06	^∗∗∗^
*HSP70-08834*	Temperature	24.666	4	6.166	22.351	3.823E-07	^∗∗∗^
	Subspecies × temperature	29.610	4	7.402	26.831	8.622E-08	^∗∗∗^
	Subspecies	250.035	1	250.035	11.061	0.0034	^∗∗^
*HSP20-04164*	Temperature	3658.750	4	914.687	40.464	2.563E-09	^∗∗∗^
	Subspecies × temperature	389.911	4	97.478	4.312	0.0112	^∗^
	Subspecies	0.547	1	0.547	7.693	0.0117	^∗^
*HSP40-06977*	Temperature	2.080	4	0.520	7.306	0.0009	^∗∗^
	Subspecies × temperature	0.706	4	0.176	2.479	0.0770	ns
	Subspecies	55.145	1	55.145	17.927	0.0004	^∗∗∗^
*HSP40-09495*	Temperature	250.373	4	62.593	20.348	8.050E-07	^∗∗∗^
	Subspecies × temperature	96.982	4	24.246	7.882	0.0006	^∗∗^
	Subspecies	0.810	1	0.810	12.559	0.0020	^∗∗^
*HSP60-02375*	Temperature	2.012	4	0.503	7.798	0.0006	^∗∗^
	Subspecies × temperature	2.038	4	0.510	7.901	0.0005	^∗∗^
	Subspecies	8.100	1	8.100	16.375	0.0006	^∗∗^
*HSP90-17621*	Temperature	48.152	4	12.038	24.334	1.924E-07	^∗∗∗^
	Subspecies × temperature	19.433	4	4.858	9.821	0.0001	^∗∗^
	Subspecies	0.892	1	0.892	6.237	0.0213	^∗^
*HSP90-25730*	Temperature	3.559	4	0.890	6.218	0.0020	^∗∗^
	Subspecies × temperature	3.010	4	0.753	5.259	0.0046	^∗∗^

**TABLE 5:** Two-way ANOVA analysis of HK, PK, and PEPCK genes expressions of *Crassostrea gigas gigas* and *C. gigas angulata* under different temperature treatments (^∗∗∗^*P* < 0.0005, ^∗∗^*P* < 0.005, ^∗^*P* < 0.05; ns, not significant).

	**Factors**	**SS**	**df**	**MS**	***F***	***P*-value**	**Significant**
	Subspecies	1.332	1	1.332	59.179	2.123E-07	^∗∗∗^
HK	Temperature	3.221	4	0.805	35.790	7.486E-09	^∗∗∗^
	Subspecies × temperature	1.482	4	0.370	16.464	4.079E-06	^∗∗∗^
	Subspecies	0.006	1	0.006	0.322	0.5768	ns
PK	Temperature	0.629	4	0.157	8.328	0.0004	^∗∗∗^
	Subspecies × temperature	0.239	4	0.060	3.164	0.0362	^∗^
	Subspecies	1.588	1	1.588	33.955	1.061E-05	^∗∗∗^
PEPCK	Temperature	3.299	4	0.825	17.630	2.437E-06	^∗∗∗^
	Subspecies × temperature	0.765	4	0.191	4.088	0.0140	^∗^

## Discussion

Studies on the impacts of temperature on the physiological functions of organisms are essential for interpreting how ocean warming influences marine resources and biodiversity. Studies on phenotypic trait variation in broadly distributed species provide a framework for descriptive characterizations of biogeographic distributions and local adaptations, which can lead to spatial differences in thermal tolerance among populations (Pörtner, 2001; Fangue et al., 2006; Pörtner et al., 2006). As a consequence of evolutionary adaptation, different thermal specializations and limitations are conserved among different acclimatized conspecific species (Bozinovic et al., 2011; Pörtner, 2012; Dowd et al., 2015). Here, we demonstrated that thermal performance divergence in relation to aerobic scope was correlated with the temperature regimes of two oyster subspecies. Additionally, we attempted to evaluate the adaptive physiological traits (metabolic rate, heart rate, and expression of thermal responsive genes) (Kassahn et al., 2009; Schulte et al., 2011) of these two subspecies to increasing temperature based on thermotolerance metrics. Overall, *C. gigas gigas* was extremely sensitive to warming conditions, whereas *C. gigas angulata*, which is found in the South China Sea, will likely be able to withstand temperature increases in the face of climate warming.

### Thermal Tolerances of the Oyster Subspecies

Evaluating thermotolerance by measuring the LT_50_ of a species to determine how species cope with thermal extremes provides an effective indicator to identify the limits of thermal tolerance (Cooper et al., 2008; Bayne, 2017). This study showed that 100% survival of *C. gigas gigas* and *C. gigas angulata* could be achieved by exposing the oysters to 37°C within 1 h, and the median upper lethal limits (LT_50_) were 42.4 and 43.7°C, respectively (Figure 2); direct heat shock at 44°C resulted in 100% mortality. In Pacific oyster, heat shock at 37°C for 1 h did not lead to any mortality, and the LT_50_ was 42.3°C for this species, which agreed with our result for *C. gigas gigas* (Shamseldin et al., 1997; Clegg et al., 1998). In other studies, the first signs of mortality at 35°C were observed when the duration was increased to 24 h, which occurred along with a high expression of apoptosis-associated genes (Zhu et al., 2016). Rajagopal et al. (2005) determined the relationship of thermal tolerance to different size groups and reported better thermal resistance of larger animals than smaller ones. Moreover, pretreated heat shock of these subspecies promoted their heat resistance, and the southern oyster had a better ability to withstand heat shock than its counterpart (Ghaffari, unpublished), which was confirmed with other prior observations in Pacific oyster (Shamseldin et al., 1997; Clegg et al., 1998). Additionally, in the pilot year of the study, individuals of these two subspecies of similar ages and sizes were reciprocally transplanted, and high mortality and a lower growth rate were found at the southern site (warmer location) than at its local site for *C. gigas gigas* (Li, unpublished). A powerful linkage between the thermal tolerance limits of oysters and the temperature of their environments make the thermal regime the most acceptable description for variation in their thermal performances; however, we cannot eliminate the possible contribution of other ecological variables (such as food availability in the sea). As a result, *C. gigas angulata* had a more moderate thermal tolerance than *C. gigas gigas*. This investigation provided baseline data for further research on thermal biology. To more fully determine the role of temperature in the relationship between physiological flexibility and thermotolerance divergence, additional studies, such as studies on the functions and interactions of thermal responsive genes, are required (Bayne, 2017).

### Thermotolerance Windows and Physiological Variation Responses

In the present study, the two oyster subspecies exhibited different thermotolerance windows in relation to their metabolic and gene expression. Here, the cardiac activities, oxygen consumption levels and HSPs of both subspecies were affected significantly under ecologically realistic rising temperatures, which was observed in similar previous studies on oyster physiological performance (Dunphy et al., 2006; Lannig et al., 2008; Giomi et al., 2016). The southern oysters, *C. gigas angulata* (warm-temperate oyster), exhibited a higher and extensive metabolic rate compared with its northern counterpart, *C. gigas gigas* (cold-temperate oyster). Similar to our result, in a common garden experiment, the southern oyster had a higher metabolic rate when exposed to sublethal heat shock (35°C), while a lower level of metabolic compensation was observed in the northern species (Li A. et al., 2017), which was also similar to other studies (Jansen et al., 2007; Rastrick and Whiteley, 2011).

The pattern of increased followed by decreased aerobic metabolic activity over periods of thermal stress was indicated in oyster (Zhang et al., 2015; Li A. et al., 2017; Li A. et al., 2018; Li L. et al., 2018). In this study, there were clear differences in the ABT that were associated with the different metabolic capabilities of these two subspecies. The ABTs were 29.22 and 33°C for the northern and southern oysters, respectively, which was consistent with the previously mentioned outcomes. In addition, the calculated *Q*_10_ values indicated no dependence of SMR on temperature above the ABTs. Moreover, the accumulation of anaerobiosis end products indicated the switch from aerobic to anaerobic mitochondrial metabolism and defined the critical thresholds (Michaelidis et al., 2005; Tagliarolo and McQuaid, 2015; Meng et al., 2018). Consequently, ABT may serve as a marker of the critical temperature at which metabolism changes for aerobic to anaerobic (Sokolova et al., 2012). This transition is supported by OCLTT models that highlight disorders in the oxygen supply, declines in aerobic scope and the onset of anaerobic metabolism at extreme temperatures (Pörtner, 2010; Sokolova et al., 2012). In line with our finding, this evidence was also reported in similar studies through key enzyme activities (*PK*, *PEPCK*), anaerobiosis end products and the expression patterns of metabolic genes (Li A. et al., 2017; Li L. et al., 2018; Meng et al., 2018). In the current study, the southern oysters exhibited a higher SMR at 32–40°C, which indicated a greater ability of this subspecies to provide sufficient aerobic energy that is required for somatic maintenance than its northern counterpart. Similar to our findings, distinct differences in the metabolic rate and expression patterns of metabolism genes indicated the better protective performance of the southern oysters when exposed to long-term heat shock (Li A. et al., 2017). Additionally, comparing the levels of anaerobic products and *PEPCK* between these subspecies, lower levels of these parameters could not be considered a marked stress response in the southern oysters. The heartbeats of the southern oysters were slow, and the anaerobic end products and gene expression levels were lower, suggesting a greater capacity of thermal insensitivity and likely metabolic depression to ensure immediate survival (Le Moullac et al., 2007; Sokolova et al., 2012).

Species-specific mechanisms of thermotolerance based on the expression of HSPs result from evolutionary adaptation to specific thermal habitats (Pörtner, 2012; Tomanek, 2014). Our results and other supported findings indicate that HSP expression is related to the upper thermal tolerances of these two subspecies. While the northern oysters exhibited higher HSP (especially HSP70) induction at intermediate temperatures (29–36°C), their southern counterparts were capable of upregulating HSP70 and HSP40 at extreme temperatures (40°C). This expression divergence may reflect the higher thermal sensitivity of the northern oysters compared with their southern counterpart, which contributes to its ability to survive under extremely high temperatures. Species variations in HSP expression patterns can reflect both the evolutionary thermal histories of species and the recent thermal acclimation conditions encountered by an organism (Hochachka and Somero, 2002; Tomanek, 2008). In our work, most of the HSPs in the northern oysters were first induced (T_on_) at 29°C, the high end of the non-stressful heat range. The maximum (T_*peak*_) was reached at 36°C for certain HSPs to provide protection up to temperatures that exceeded the thermotolerance limits of the oysters (T_off_), when HSP synthesis was heat-inactivated. In contrast, these temperature set-points were higher in the southern oyster subspecies, which inhabits a warmer environment. Comparative studies such as that of Tomanek (2008) and the present study cannot directly link the patterns of HSP expression and overall thermal tolerance. However, these studies provide evidence of the underappreciated variety of HSP expression patterns association with differences in whole-organism thermal tolerance and their possible role in the establishment of biogeographical patterns. Overall, the occurrence of divergent heat shock responses can express the distinct evolutionary backgrounds of these subspecies and may play important roles in setting their biogeographic distributions and thermotolerance limits. In temperate regions, more cold-adapted species generally show HSP upregulation at lower temperatures than their warm-adapted counterparts, as was shown in mussels (genus *Mytilus*) (Tomanek, 2014). The transcriptomic responses of tidal oysters to thermal stress demonstrated fine-scale vertical adaptive divergence in which intertidal oysters exhibited high HSP upregulation and metabolic rates compared with subtidal populations (Li A. et al., 2018). Moreover, intraspecific comparisons of *C. gigas gigas* under acute heat shock (35°C) revealed that in a highly variable environment, the northern population has more thermal resistance via adaptive physiological plasticity (Li L. et al., 2018). In this study, distinct upper thermal tolerance was demonstrated. These two oysters exploited strategies of metabolic activity with their specific onsets of heat shock response, which occurred close to their breakpoint temperatures. After that time, their aerobic scopes become constrained. In this study, the critical thermal maxima were significantly different between these two oyster subspecies, which were 28–32 and 32–36°C for the northern and southern oysters, respectively. Unfortunately, laboratory studies are strongly dependent on the rate of increase, and laboratory simulation can generally only be considered an approximation of the real thermal limits experienced by individuals under natural conditions (Stillman, 2002; Rastrick and Whiteley, 2011; Dong et al., 2017). Such an experiment allows the possibility of a phenotypic response that influences future physiological performance (Bayne, 2017).

Adaptive physiological and molecular mechanisms that enable species to tolerate heat stress are energetically demanding (Paschke et al., 2018). When animals are faced with moderate stress, metabolism and ATP turnover accelerate to provide the required maintenance cost and compensate for the increased energy demands for the protection of physiological functions (Paschke et al., 2018). During extreme stress, aerobic metabolism is impaired, and aerobic metabolism switches to anaerobic metabolism to compensate for declines in aerobic energy stores (Pörtner, 2012; Sokolova et al., 2012); however, this type of metabolism is less efficient than aerobic metabolism and is followed by end-product accumulation and intracellular acidosis (Bayne, 2017; Meng et al., 2018). Overall, physiological divergence across latitudinal gradients reflects the result of adaptive differences and can also be caused by neutral variation and various types of flexibility (Somero, 2010; Dowd et al., 2015). According to climate variability hypotheses, higher latitude species have broader thermotolerances to cope with their wide climatic windows (Stillman, 2002; Bozinovic et al., 2011). However, in this work, the southern oyster subspecies (warm-adapted) displayed a higher thermotolerance capacity at low latitudes compared with its northern counterpart (cold-adapted) at higher latitudes. Moreover, it has been proposed that warm-adapted species need a greater degree of thermotolerance to be resistant to a thermally stressful environment (Yampolsky et al., 2014; Stuart-Smith et al., 2017). Such findings support the idea that oysters that settle in thermally stressful environments may evolve high physiological flexibility to thermotolerance (Rastrick and Whiteley, 2011; Li L. et al., 2018). Our results provide further evidence of thermotolerance divergence in these two oyster subspecies; furthermore, the results indicate that the ability to adjust the physiological performance of oysters inhabiting thermally stressful environments might be critically important in the face of global warming (Angilletta, 2009; Rastrick and Whiteley, 2011; Schulte et al., 2011).

## Conclusion

These oyster subspecies exhibited different levels of physiological performance that were associated with geographic differences in their environmental temperatures. The southern oysters exhibited better aerobic performance under heat stress conditions and may be able to adjust their thermotolerance limits. Additionally, differences in the HSP gene expression patterns were linked to the distinct thermal environments of the oysters. In this work, based on the physiological and molecular metrics of heat response, it is clear that these oyster subspecies have distinct thermal optima. However, physiological studies on the capabilities of thermal acclimatization are essential to interpret biological responses to climate change.

## Data Availability

All datasets generated/analyzed for this study are included in the manuscript and the Supplementary Files.

## Author Contributions

GZ and LL designed and supervised the research. HG performed the laboratory experiment, data analyses, and drafted the manuscript. WW collected the animal. HG, AL, GZ, and LL contributed to the interpretation of the results and substantial revision of the manuscript. All authors contributed to approving the final version of the manuscript for publication.

## Conflict of Interest Statement

The authors declare that the research was conducted in the absence of any commercial or financial relationships that could be construed as a potential conflict of interest.

## Footnotes

1 https://oceandata.sci.gsfc.nasa.gov/

2 www.presense.de
