# Supplementary material for: Thermotolerance Divergence Revealed by the Physiological and Molecular Responses in Two Oyster Subspecies of Crassostrea gigas in China
Source: Front Physiol. 2019 Sep 10;10:1137. doi: 10.3389/fphys.2019.01137 (PMC6746976; doi:10.3389/fphys.2019.01137)
Supplement: Supplementary file 1 [file Data_Sheet_1.docx]

Supplementary Material

**Thermotolerance divergence revealed by the physiological and molecular responses in two oyster subspecies of *Crassostrea gigas* in China**

**Hamze Ghaffari, Wei Wang, Ao Li, Guofan Zhang*, Li Li***

*** Correspondence:** Li Li, [lili@qdio.ac.cn](mailto:lili@qdio.ac.cn). Guofan Zhang, [gfzhang@qdio.ac.cn](mailto:gfzhang@qdio.ac.cn)

## Supplementary Figures


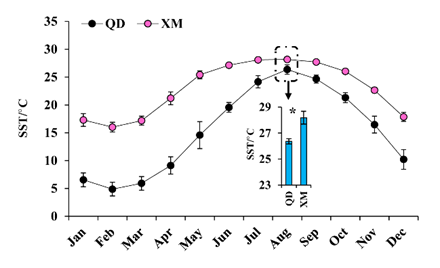


**Supplementary Figure 1.** Average monthly of sea surface temperature (SST) within 30 kilometers of the Northern (Qingdao; QD) and Southern (Xiamen; XM) sampling sites during the last 18 years (from 2000 to 2018); asterisk indicates a significant differences (*p* < 0.05).

**
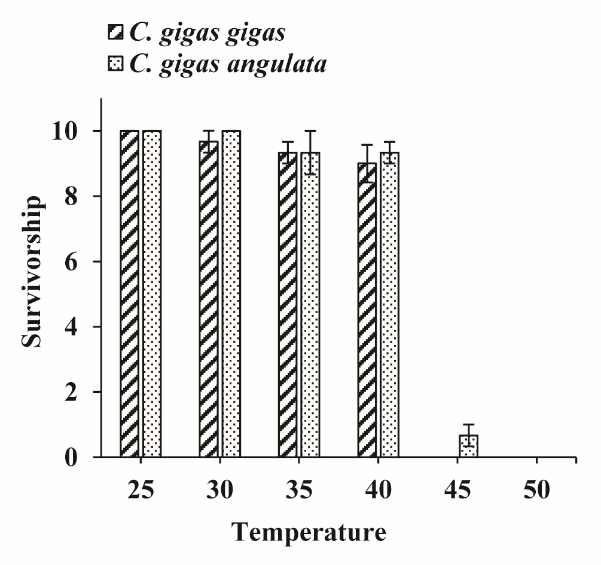
**

**Supplementary Figure 2.** Preliminary heat shock treatment indicated the survival temperature range for the *Crassostrea gigas gigas* and *C. gigas angulata* 7 days after acute heat shock.

**
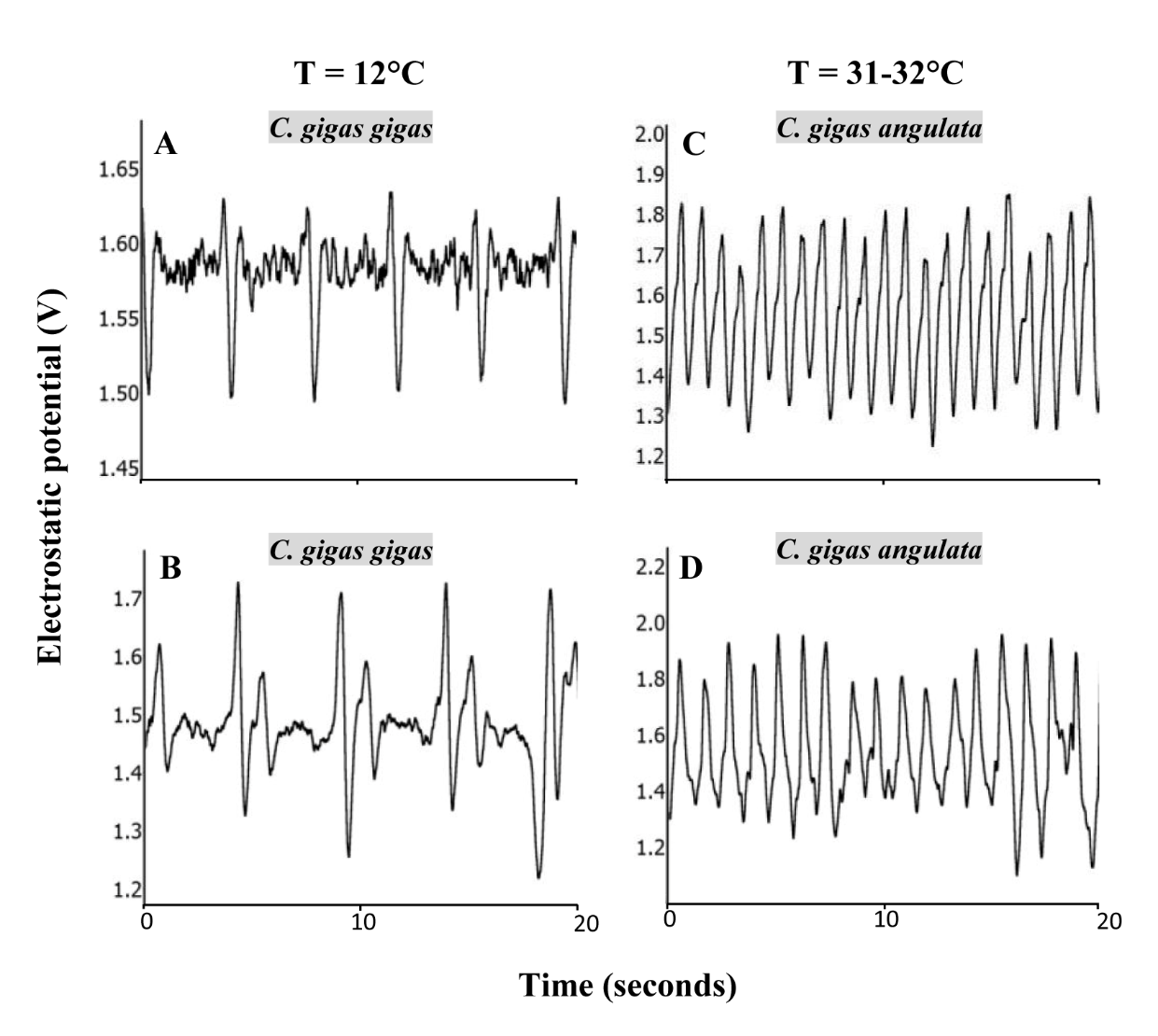
**

**Supplementary Figure 3.** Representative examples of variation in the heart rate traces recorded after 55 or 60 min constant at the beginning (12 °C) for *Crassostrea gigas gigas* (A) and *C. gigas angulata* (B); and heating (31–32 °C) for *Crassostrea gigas gigas* (C) and *C. gigas angulata* (D). Time scale is 15 s and the different scale of the y-axes depending on subspecies.

## Supplementary Table

| **Gene name** | **Abbreviation** | **Primer** | **sequence (5'-3')** | **Size (bp)** | **E_a_** | **E_b_** |
| --- | --- | --- | --- | --- | --- | --- |
| Heat shock proteins | HSP70-02823 | F | 5'-GCTGTGGCTTATGGAGCTGCTG-3' | 105 | 1.11 | 1.09 |
|  |  | R | 5'-TCCTGCCGTTTCAATGCCCAAA-3' |  |  |  |
|  | HSP70-15492 | F | 5'-ACCGTGGAGTCAACCCTGATGA-3' | 102 | 0.91 | 0.97 |
|  |  | R | 5'-CGTCCAACAGCAGAAGGTCACC-3' |  |  |  |
|  | HSP70-08834 | F | 5'-CCAGAACGACAACAACAGACTCTCA-3' | 108 | 0.92 | 0.95 |
|  |  | R | 5'-TTGGCTTCAACCTTCTCCTTCACTT-3' |  |  |  |
|  | HSP20-04164 | F | 5'-CGCCATTACGGACGGCAAGAA-3' | 105 | 0.96 | 1.05 |
|  |  | R | 5'-ACGGTAATGTGGTCAGGCTCGA-3' |  |  |  |
|  | HSP40-09495 | F | 5'-GGAGGAGACGACCCGTTTGCTA-3' | 103 | 0.96 | 0.99 |
|  |  | R | 5'-GTTGCCCGCCGAAATGGAACA-3' |  |  |  |
|  | HSP40-06977 | F | 5'-CACATTTCCAGAAGAAGGCGACCA-3' | 128 | 1.02 | 0.98 |
|  |  | R | 5'-GAACCTTGGCAGTGTGGATCAGATT-3' |  |  |  |
|  | HSP60-02375 | F | 5'-TTGCTGACTCGGGCTGTACTGT-3' | 103 | 0.99 | 0.97 |
|  |  | R | 5'-GACATCAGGCGGACCACTAGGA-3' |  |  |  |
|  | HSP90-17621 | F | 5'-CGAGGAACAGAAGGCTGAGTACGA-3' | 107 | 1.00 | 0.98 |
|  |  | R | 5'-AAGGAGATGTCACCAGACGGTTAGA-3' |  |  |  |
|  | HSP90-25730 | F | 5'-GGCAAGGACGACTACGAGAAGTTC-3' | 130 | 0.94 | 0.96 |
|  |  | R | 5'-CCGTGTCAGAGTTGGAGGAGTAGAA-3' |  |  |  |
| Hexokinase | HK | F | 5'-TCCTCCACGAAGCAATC-3' | 267 | 0.99 | 1.02 |
|  |  | R | 5'-TGACAAAGTCCAGACACCC-3' |  |  |  |
| Pyruvate kinase | PK | F | 5'-GCCATCATTGTTATCACTACCT-3' | 109 | 0.98 | 0.99 |
|  |  | R | 5'-ACTGACGAGCTGTCTGTTCTAT-3' |  |  |  |
| Phosphoenolpyruvate carboxykinase | PEPCK | F | 5'-CTGGGCTGGGCGACATT-3' | 153 | 0.99 | 0.99 |
|  |  | R | 5'-GCTTCTCCTGGGCGTTG-3' |  |  |  |
| Elongation factors | EF1α | F | 5'-AGTCACCAAGGCTGCACAGAAAG-3' | 199 | 0.99 | 1.02 |
|  |  | R | 5'-TCCGACGTATTTCTTTGCGATGT-3' |  |  |  |
|  |  |  |  |  |  |  |

**Supplementary Table 1.** Primer sequences and their size of genes used for the real-time PCR in *Crassostrea gigas gigas* and *C. gigas angulata* (E_a_ and E_b_ are PCR efficiency for *C. gigas gigas* and *C. gigas angulata*, respectively).

| Subspecies | Logistic regressions | Third order regressions | *P*-value | *r*^2^ (LGR) | *r*^2^ (3th order) | *r*^2^ differences (3th order-LGR) |
| --- | --- | --- | --- | --- | --- | --- |
| *C. gigas gigas* | *y*=59.053*x*˗91.115 | *y*=˗0.528*x*^3^+61.562*x*^2^˗2392.8*x*+31130 | <0.001 | 0.896 | 0.997 | 0.101 |
| *C. gigas angulata* | *y*=60.22*x*˗93.621 | *y*=˗0.701*x*^3^+83.697˗3328.8*x*+44224 | <0.001 | 0.885 | 0.994 | 0.109 |

**Supplementary Table 2.** Logistic (LGR) and Third order regression equations and model comparisons of *r*^2^.
